# Occupant behavior, thermal environment, and appliance electricity use of a single-family apartment in China

**DOI:** 10.1038/s41597-023-02891-9

**Published:** 2024-01-11

**Authors:** Chuang Wang, Xiaoyan Li, Wei Sun, Jingjing An, Shufang Gao

**Affiliations:** 1 School of Environment and Energy Engineering, Beijing University of Civil Engineering and Architecture, Beijing, 100044 China; 2 Xicheng District People’s Government of Beijing Municipality, Beijing, 100032 China

**Keywords:** Energy and behaviour, Energy management

## Abstract

The household is the basic unit of a residential community or building. High-resolution, long-term open data are necessary to help study residential electricity consumption, smart home technologies, and electricity flexibility technologies at household level. This paper introduces an IoT-based data collection platform (IDCP) consisting of gateways, sensors, and cloud servers. This platform can collect data on the occupant presence, indoor environment, window-opening states, and appliance electricity consumption simultaneously. This study deployed the IDCP in a single-family apartment in Beijing, China, and compiled a dataset, namely, CN-OBEE, including data on the per-minute occupant behavior, thermal environment, and appliance electricity use of the apartment for an entire year (from May 31, 2021, to May 31, 2022), and hourly meteorological data collected by the nearest national weather station during the same period. This dataset is the first detailed and publicly available occupant behavior and electricity use dataset for Chinese homes. As a regional feature, the dataset compiled by this study includes window-opening behavior and the use of split air conditioners (ACs).

## Background & Summary

In recent decades, global consensus has aligned with the imperative of fostering low-carbon development, and researchers have made significant efforts and innovations toward achieving this objective. Among them, electricity flexibility and demand response technology can guide users toward maximizing the utilization of clean electricity produced by solar or wind energy, improving power grid stability, and reducing carbon emissions^1^. Buildings are major energy consumers, accounting for more than 34% of the total energy demand and approximately 37% of CO_2_ emissions globally, while residential buildings comprise over 50% of this portion^2,3^. Therefore, it is essential to incorporate residential building loads into demand response schemes.

Smart home technologies allow more flexible electricity usage for residential buildings, making them more amenable to participating in demand response than ever before^4^. From the perspective of electricity flexibility, domestic appliances in residential buildings can be divided into schedulable and non-schedulable devices. Typically, non-schedulable loads, such as lights, TVs, and refrigerators, are not easy to shift without affecting the normal use of occupants. For example, it is unreasonable for occupants to turn off the lights at night. In contrast, schedulable loads, including buffered appliances such as air conditioners (ACs) and water heaters, and postponable appliances such as rice cookers and washing machines, can be shifted easily^1^. Typically, schedulable appliances are high-power devices and considered as flexible resources, whereas non-schedulable appliances, such as lights and all types of low-power electrical equipment, are not well-suited for participation in demand response^5^.

The household is the basic unit of a residential community or building. To determine the quantity of electricity flexibility and find a way to shift loads at household level, it is necessary to know the existing load characteristics and understand the actual needs of households before they participate in power grid interaction. This knowledge and understanding rely on detailed data measured per household to the extent possible. Unlike commercial and industrial buildings, residential buildings have more occupant behavior-related appliances. Owing to private ownership, appliance use is freely controlled by the occupants, and the diversity of occupant behavior is an important reason for the difference in building energy use^6,7^. For example, the electricity use of appliances depends on how long they are switched on, and their on/off state is typically related to the occupancy state (presence or absence). Split ACs are commonly used in Chinese residential buildings, contributing to the electric power peak in summer, and are important flexibility resource. Moreover, their usage is closely related to the indoor/outdoor thermal environment and window-opening behavior^8,9^. A typical behavior pattern is that the AC in a room is turned on only when the room is occupied and the room temperature reaches a certain level^8,10^. In China, window-opening is the most effective and convenient natural ventilation method for improving the indoor environment quality^11^, and is typically related to the use of split ACs, that is, occupants may close the windows or leave them open when turning on ACs, which affects the power consumption of ACs. In other words, residential energy use is affected by many factors and has strong intrinsic relationships. A comprehensive dataset combining such data as occupant behavior, the indoor and outdoor environment, and appliance electricity use will help in better understanding intrinsic relationships in residential buildings.

High-resolution, long-term *in-situ* monitoring datasets are strongly needed to study residential building energy. For example, Ren *et al*.^12^ established several typical usage patterns for household appliances by measuring the power of appliances in a single-family residence. Jin *et al*.^13^ verified a TV usage behavior model using a single-family residence’s long-term per-minute occupancy and television power data. Lu *et al*.^14^ proposed an AC turning-on behavior model based on room-level occupancy and temperature data. Based on the monitoring of water temperature, indoor temperature, and electric power, Tejero-Gomez *et al*.^15^ designed an energy management system for electric heaters, and tested it in a single-family residence. However, the collection of such data is often time-consuming and challenging, owing to cost and privacy concerns. Traditional building energy monitoring relies on many wired sensors and a central monitoring system, which are typically available only for new buildings. Therefore, independently packaged meters are generally used for existing residential buildings. However, collecting occupant behavior and appliance electricity use data is not convenient owing to the size, cost, and required amount of meters^8,12,16^.

To date, few public residential datasets have included detailed energy use and occupant behavior data. The details of existing publicly available residential datasets are shown in Supplementary Table 1^17–33^. These datasets have their own application purposes and focus on different data elements. The REDD^17^, BLUED^18^, FIRED^19^, DEDDIAG^20^, ENERTALK^21^, UK-DALE^26^, SustDataED^28^, REFIF^29^, EMBED^30^, and fIEECe^31^ datasets focus on the high-frequency electricity consumption data of the entire house and individual appliances, aiming to investigate building electricity disaggregation and energy use models. Typically, these datasets have a sampling rate in seconds or milliseconds and a short collection period ranging from a few days to dozens of days. The BLUED^18^ dataset only labels the switching state of individual appliances and does not include energy consumption data. The DEDDIAG^20^ and ENERTALK^21^ datasets include data on many electrical appliances; however, the appliance types are relatively few and include only non-schedulable appliances such as kitchen, cleaning, and entertainment appliances, while schedulable appliances such as ACs are not included. Several datasets^23–25^ focus on occupancy data and are intended for use in building occupancy detection analysis. The Global Building Occupant Behavior Database^25^ focuses on occupant behavior, and includes four *in-situ* residential datasets: Dataset-8^34^, which is a dataset of the indoor and outdoor environment and window status over three months from one Canadian residential building; Dataset-11^35^, which is a dataset of occupant presence over one year from three American houses; Dataset-13^36^, which is a dataset of the outdoor environment and daily AC energy consumption over one year from one Polish residential building; Dataset-15, which is a dataset of the outdoor environment and window status over six months from four Chinese apartments. A few datasets^27,32,33^ focus on building energy use, but lack occupant behavior and indoor environment data.

Public residential datasets generally include inadequate data on the occupant behavior, thermal environment, and electricity use, and have inadequate balance between the measurement period and sampling frequency, weakening their application potential in residential energy flexibility analysis. Particularly, there is a lack of data considering the occupancy, window-opening behavior, and electricity of schedulable appliances (split ACs) simultaneously. One important reason for the lack of such data is the lack of small, portable, low-cost meters for various measurements in residential buildings^16^. With the popularity of Internet-of-Things (IoT) devices in recent years, it has become possible to use smart IoT devices to carry out convenient, low-cost, and long-term comprehensive monitoring of residential occupant behavior and energy use.

This paper introduces an IoT-based Data Collection Platform (IDCP) consisting of gateways, sensors, and cloud servers. This platform can simultaneously collect and upload data on occupant presence, the indoor thermal environment, window-opening behavior, and appliance electricity consumption. The IDCP was built based on smart IoT devices and cloud services, and has the following advantages: 1) tiny wireless sensors consume little space and have little impact on home appearance; 2) low-power sensors support long-term measurement and do not require frequent battery replacement; 3) automatic, continuous, and remote real-time data collection; 4) the devices have low cost. This study deployed the IDCP in a single-family apartment in Beijing, China, and compiled a dataset, namely, CN-OBEE^37^, which includes per-minute data on the occupant behavior, thermal environment, and appliance electricity use of an apartment over an entire year (from May 31, 2021, to May 31, 2022), and hourly weather data for the same period, which were collected from the nearest national weather station. The selected family home is representative of Beijing’s urban family residences. As one of the regional features in the dataset, the dataset compiled by this study covers window-opening behavior and the use of split ACs, which is currently the mainstream cooling method in Chinese residential buildings. To the authors’ knowledge, the CN-OBEE dataset^37^ is the first publicly available and detailed occupant behavior and electricity consumption dataset of Chinese homes, contributing to the regional diversification of globally available datasets.

The features of the CN-OBEE dataset^37^ are as follows:

- This study focused on most Chinese homes’ appliances, including schedulable appliances, such as split ACs, water heaters, washing machines, and rice cookers, and non-schedulable appliances, such as fridges and TVs.
- The data were collected over one year, and the sampling frequency is one minute after processing.
- The dataset was automatically, continuously, and remotely collected using the IDCP.
- The dataset consists of data on room-level indoor environmental parameters, window-opening states, occupancy presence, appliance electricity use, and outdoor meteorological conditions from the nearest national weather station.

This dataset can be directly used to analyze the quantitative and temporal characteristics of the electricity loads of various appliances (e.g., power peaks, seasonal changes, differences between weekdays and weekends, dependence on occupancy and indoor/outdoor environment, correlation between the use of various appliance), and is the basis for investigating electricity flexibility at household level. In addition, the dataset can be used for (1) electricity load shape analysis to reveal the whole-family and appliance-level demand profiles^12,13^; (2) electric power forecasting using statistical or machine-learning algorithms^38^; (3) physical-based or data-driven modeling and validation of occupancy, window-opening behavior, and energy-use behavior for appliances^6,39^; (4) analytics on the indoor thermal environment and indoor thermal comfort^40^. Although the dataset represents a single household, it is still an important initial contribution to this research field owing to its comprehensiveness and uniqueness.

## Methods

### Description of apartment and family

In this study, data were collected from a single-family apartment that was recruited and screened by the research team. The recruitment was aimed at college students, and information such as family structure, income, housing type, and appliance type was collected through an online questionnaire. The family considered in this study was recruited as a representative family, because it is a middle-class nuclear family residing in a privately-owned, well-maintained apartment with a wide variety of modern appliances, which is representative of Beijing’s urban family residences. The family’s participation in this study was completely voluntary and monetary compensation was not provided.

**Fig. 1:**
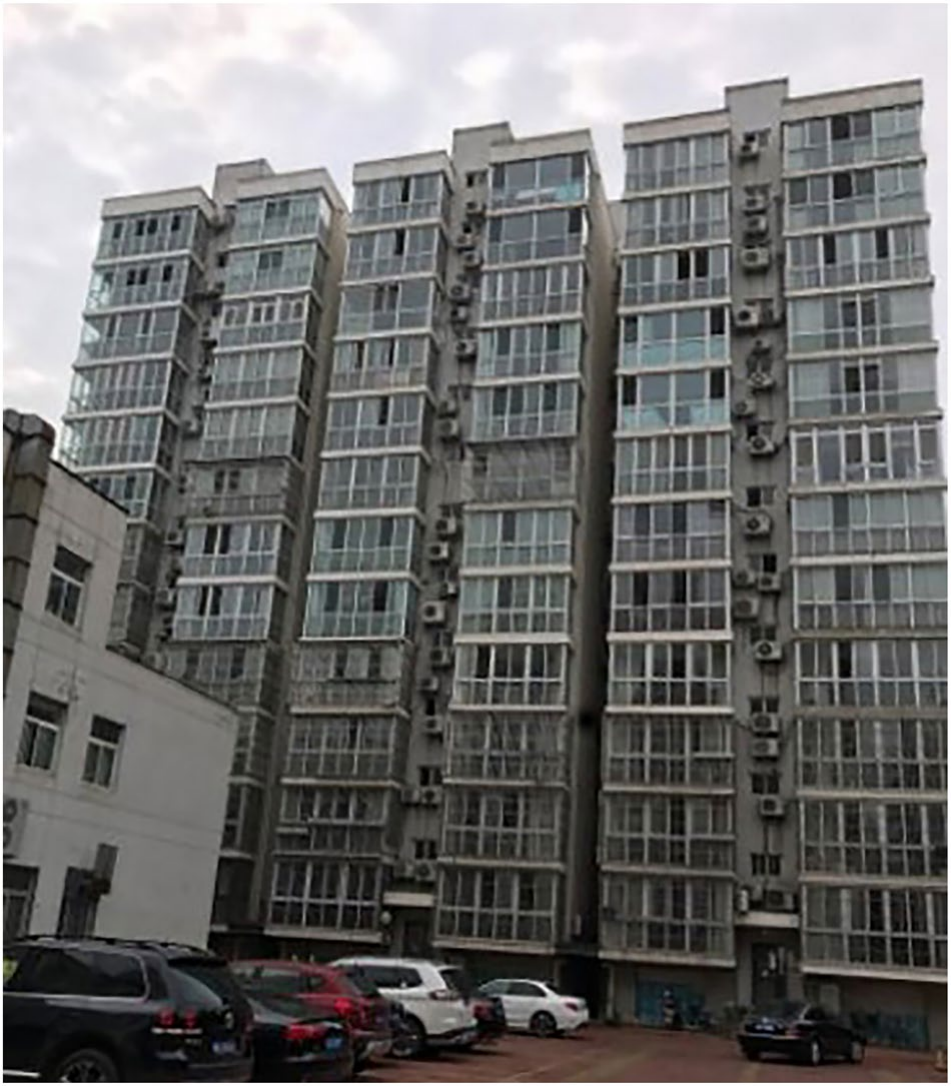
Building appearance (south elevation).

The apartment building has the most common housing form in urban China, and is located in Miyun District, Beijing. The building (Fig. 1) was constructed in 2005 and consists of 13 floors and 48 households.

**Fig. 2:**
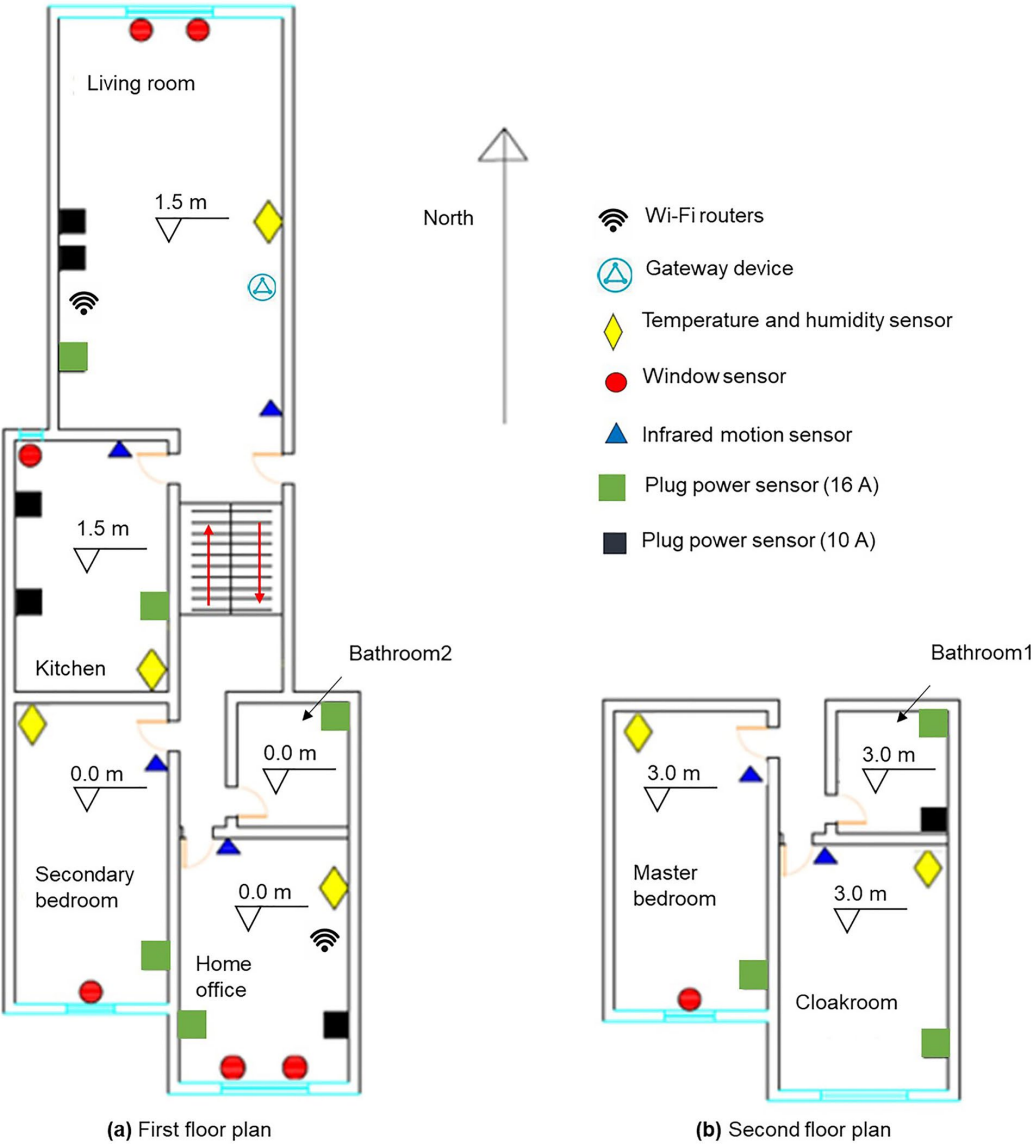
Floor plans of apartment and sensor locations.

The apartment is a two-story duplex located at the middle floor of the building, and has an area of 172.73 m^2^ and floor height of 3 m. The apartment has eight rooms, including a master bedroom, secondary bedroom, cloakroom, home office, living room, kitchen, and two bathrooms. The apartment’s floor plans are shown in Fig. 2.

The apartment is used by a family of three: a middle-aged couple and their adult offspring. The couple occupies the master bedroom and works only on weekdays. Their adult offspring is a full-time student at a local university and occupies the secondary bedroom, but often resides at school instead of living at home. To assist in collecting and sharing the CN-OBEE dataset^37^, the family kindly agreed to install metering devices in their apartment and allow public access to the measured data. They acknowledged that their typical living habits were not affected by the study. Unnecessary or private information about the family was removed from the final dataset by pre-processing.

### Data collection

The IDCP is currently deployed for monitoring, and various sensors are installed throughout the apartment to collect data on the occupancy presence, indoor thermal environment, window-opening state, and appliance electricity usage. The measurand and platform details are described in the following section.

In addition, hourly outdoor weather data, including the dry-bulb temperature, relative humidity, atmospheric pressure, wind speed, wind direction, ground temperature, horizontal total solar radiation intensity, and horizontal diffuse solar radiation intensity, were obtained from the national weather station in Miyun District.

### Measurands and sensor deployment

The measurands in the apartment were obtained using smart IoT sensors from a brand manufacturer with industrial-grade reliability and accuracy. The deployment layout of all sensors is shown in Fig. 2. Table 1 lists the sensors installed in each room. The measurands were divided into four categories as described below.

**Table 1:** Number of sensors in each room.

Room Sensor		Master bedroom	Secondary bedroom	Cloak room	Home office	Living room	Kitchen	Bath room1	Bath room2
Temperature and humidity sensor		1	1	1	1	1	1	N/A	N/A
Infrared motion sensor		1	1	1	1	1	1	N/A	N/A
Window sensor		1	1		2	2	1	N/A	N/A
Plug power sensor	16A	1	1	1	1	1	1	1 (Water heater)	1 (Water heater)
	10A	N/A	N/A	N/A	1 (Computer)	2 (TV, Kettle)	2 (Rice cooker, Fridge)	1 (Washing machine)	N/A

#### Indoor thermal environment

The dry-bulb temperature and relative humidity of the six main rooms (master bedroom, secondary bedroom, living room, home office, cloakroom, and kitchen) were monitored. To measure the mean indoor thermal environment more effectively, temperature and humidity sensors were placed in the middle or corner of a wall at the height of 1.5 m, and away from the heat source. In addition to temperature and humidity, the air pressure of each room was also monitored by these sensors to assist in calculating the humid air’s thermophysical properties and analysing the natural ventilation between the rooms.

#### Occupant presence

The occupancy state (presence or absence) in the six main rooms was monitored by infrared motion sensors mounted near the door of each room and facing the occupant activity area. Because the rooms are not very large, one motion sensor can cover almost the entire room without dead corners. Motion is detected when someone enters, leaves, or moves in a room. Because the family has no pets, the infrared motion sensors only detected human movement.

#### Window-opening state

The open or closed state of all operable windows in the apartment was monitored using magnetic switch sensors. The window sensor consists of two parts: a primary unit and a magnet. These sensors were mounted along the edges of the fixed border and movable part of each window, respectively, and aligned with each other. The window-open or window-closed state was detected by considering the distance between the primary unit and the magnet. When a window was opened or closed, the action was detected and recorded. For windows with two movable parts, a window sensor was installed on each part for independent monitoring. Because the cloakroom windows are always closed, a window sensor was not installed there.

#### Appliance electric power

The use of major electrical appliances (six split-type ACs, one computer, one TV, one kettle, one rice cooker, one fridge, one washing machine, and two water heaters, amounting to a total of 14 appliances) were monitored. The six ACs were located in the six main rooms. The computer was in the home office on the first floor, which is typically used by the family’s offspring. The TV and electric kettle were located in the living room. The rice cooker and fridge were located in the kitchen. The washing machine was located in the bathroom on the first floor, and the two water heaters were located in the upstairs and downstairs bathroom, respectively. Each appliance was equipped with a plug power sensor to monitor the operating power and cumulative electricity consumption. The plug sensor had ultra-low standby power consumption (approximately 70 mW), which has minimal impact on power measurement.

#### Sensors

All smart sensors were lightweight and compact, and occupied minimal space when installed. The temperature and humidity sensor, infrared motion sensor, and window sensor were powered by built-in batteries, while the plug sensor drew power from a standard socket. Owing to their excellent low-power design, sensors using new button batteries can operate continuously for over a year. Smart sensors only support wireless communication and have no data storage function. Unlike traditional sensors, which sample data at regular intervals, smart sensors are designed to generate data when the cumulative change of the sensing status exceeds a pre-set threshold or when there is no change beyond the threshold during a long pre-set period. The threshold and period are pre-set in the firmware by the manufacturer and cannot be configured by users. Once sampled data are generated, the sensors immediately report the data to the cloud through the data collection platform developed by this study.

The manufacturer’s technical specifications (https://developer.aqara.com/console/equipment-resources) for each sensor type (exposed view, measuring variables, data unit, sensor range and accuracy, and reporting mechanism) are summarized in Table 2.

**Table 2:** Technical specifications of IoT Sensors.

Sensor Type	Exposed View	Measuring Variables	Data Unit	Sensor Range and Accuracy	Sensor Reporting Mechanism
Temperature and humidity sensor		Dry-bulb temperature	°C	Temperature: 20 °C–50 °C, ± 0.3 °C; Humidity: 0%–100%, ± 3%; Air pressure: 30 kPa–110 kPa, ± 120 Pa.	1) Temperature: report when the change exceeds ± 0.5 °C, or report along with the humidity. 2) Humidity: report when the change exceeds ± 6%, or report along with the temperature. 3) Periodic reporting if no change is detected.
		Relative humidity	%		
		Air pressure	Pa		
Window sensor		Window state	0/1	The maximum sensing distance of 22 mm	Report when a state change is detected. When the magnet moves toward the primary unit, it reports 0 (closed); when the magnet moves away from the primary unit, it reports 1 (open).
Infrared motion sensor		Occupant presence	0/1	Infrared horizontal detection distance 7 m, detection angle 170°	Report “1” when occupant motion is detected.
Plug power sensor (16 A)		Power (air conditioner and water heater)	W	Max 4000 W, ± 0.01 W	Report when the change exceeds 3% or 5 W, or periodic reporting if no change is detected.
Plug power sensor (10 A)		Power (other electrical appliances)	W	Max 2500 W, ± 0.01 W	Report when the change exceeds 3% or 5 W, or periodic reporting if no change is detected.

### IoT-based data collection platform

**Fig. 3:**
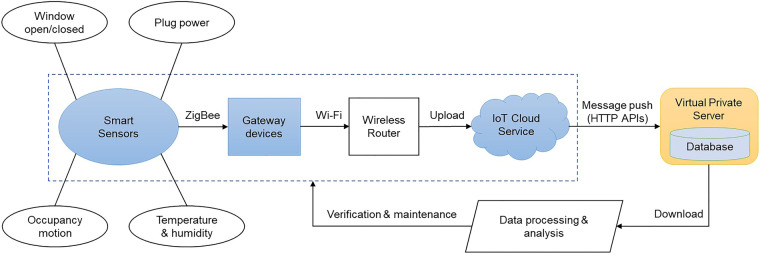
Schematic of IDCP.

A schematic of the IDCP and data flow process is shown in Fig. 3. Smart sensors, gateway devices, and cloud services are standard configurations of mainstream IoT smart home systems in the current market. The components used in this study belong to the same IoT manufacturer brand. Each smart sensor was connected to a gateway through the ZigBee protocol, while the gateway was connected to a wireless router through the Wi-Fi protocol. One gateway device and two Wi-Fi routers were used to ensure complete signal coverage. Notably, the plug power sensor (16 A), which is referred to as the AC companion (https://www.aqara.com/prodCenter), is an integrated device containing a power sensor and a gateway directly connected to a Wi-Fi router. Through the gateways and wireless routers, the successive sensing data of the sensors are uploaded and stored on the manufacturer’s cloud server. Typically, the data flow in a closed platform built by the manufacturer and can only be viewed through specific mobile apps. This study selected a specific IoT manufacturer because they provide an open cloud service and have released RESTful HTTP APIs for remote calls by authorized third-party applications, thus enabling device status queries, message pushes, and other functions. The message push service allows the reporting of real-time data collected by the sensors to a third-party server. Hence, this study built a virtual private server and an authorized app to accept the pushed messages, sort out the target data, and store the data in a SQLite database. Then, the single-file database can be downloaded for processing and analysis. Moreover, issues during the entire data collection process can be identified through real-time data analysis, and solved in time.

The advantages of the IDCP are as follows: 1) tiny wireless sensors occupy minimal space and have little impact on home appearance; 2) low-power sensors support long-term measurement and do not require frequent battery replacement; 3) automatic, continuous, and remote real-time data collection is achieved; 4) the devices have low cost. Therefore, this platform is convenient for large-scale deployment and the long-term monitoring of buildings. During deployment in a single-family apartment, the platform has been running stably for approximately two years.

### Data pre-processing

This section describes the data pre-processing steps required to obtain the dataset from the SQLite database. The database stores the raw timestamp records of the indoor thermal environment, occupant presence, window-opening state, and appliance electric power, which comes in at varying intervals, as mentioned above. The pre-processing tasks include the regularization of irregular time series data and identification and handling of missing data.

### Regularization

**Fig. 4:**
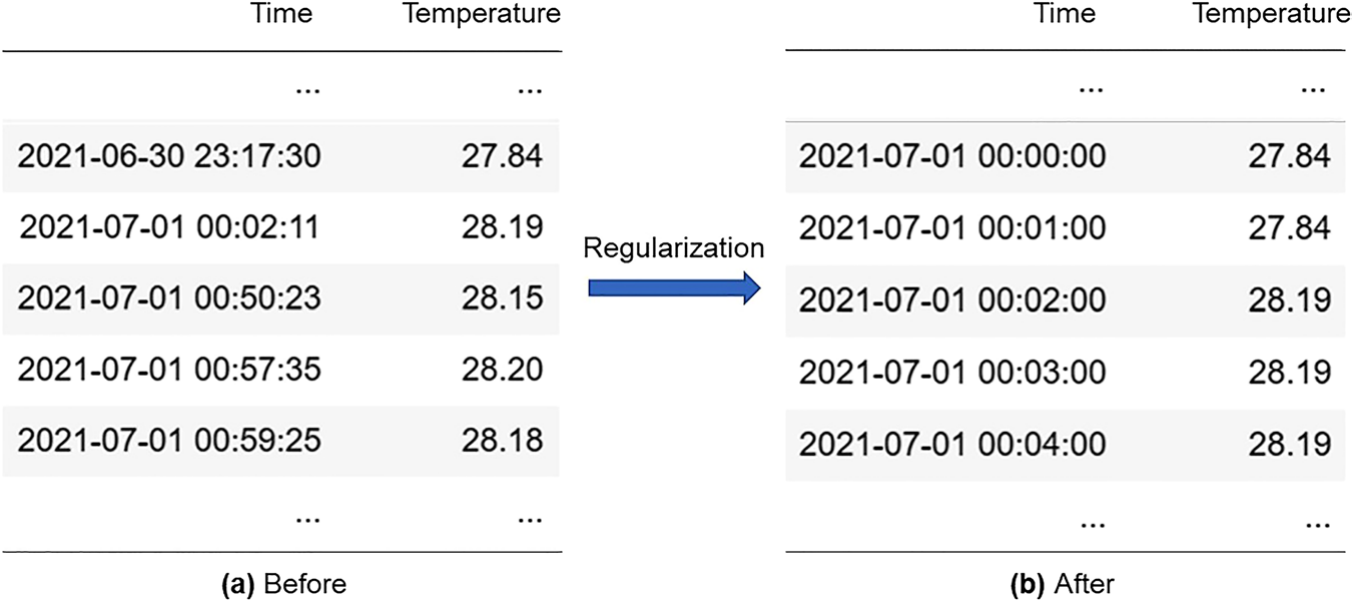
Regularization of temperature time series.

The raw time series data reported by the smart sensors have irregular time intervals, as shown in Fig. 4(a), which is inconvenient for display and analysis. Therefore, the irregular time series data must be converted to regular time series data using a unified starting time, ending time, and time interval. This study used a one-minute interval to maintain high temporal resolution. The regularized timestamps follow the date-time format (Year-Month-Day Hour: Minute: 00), in the UTC + 08:00 time zone (Beijing Time). Figure 4 shows an example of temperature time-series regularization.

The following strategies are used to calculate temporal values at one-minute intervals, fill the “normal” gaps caused by the sensor-reporting mechanism, and merge multiple data records corresponding to the one-minute interval.

- For temperature and humidity sensors, window sensors, and power sensors, the resampled value at one minute is the average of the data records occurring in that minute (from the 0^th^ second to the 59^th^ second); the gap between every two records is filled with the value recorded at the last valid timestamp (forward propagation).
- For occupancy motion sensors, the resampled value for a given minute is 1 if any motion record occurs; gaps are filled with zeros, because an algorithm that can effectively interpolate presence data does not exist. This study retains the status quo for further research in the future.

### Handling of missing data

In addition to the normal gaps caused by the sensor-reporting mechanism, there are two additional gap types in the raw dataset: 1) gaps caused by network interruptions, including lost connection between the sensors, gateways, and Wi-Fi router, and the router being powered off; 2) gaps caused by occupant habits, such as unplugging the sensors of appliances that are unused (AC, rice cooker).

The following gaps are caused by the occupants’ unplugging habits: 1) the AC power sensor is unplugged when the AC is not in use, resulting in data gaps but not missing data. These gaps were filled with zeros to ensure that the AC is not in use; 2) the plug of the rice cooker is often inserted when the appliance is in use and pulled out when the appliance is not in use. The plug often needs to be removed from the power sensor, and the sensor is occasionally removed with it, which causes inconvenience to the occupants. The occupants unplug the sensor to avoid trouble, resulting in missing data. These gaps are deleted in the regularized data.

Gaps caused by network interruptions are deleted in the regularized data. The rules for determining missing data are as follows:

- Data are considered to be missing if the temperature and humidity sensors have not reported data for 12 hours.
- Window sensors and infrared motion sensors report data when the window state changes and occupant motion is detected, respectively; therefore, the online status of the sensors is considered to determine whether data are missing. Data are considered to be missing when the sensors are offline.
- Data are considered to be missing if plug power sensors have not reported data for 36 hours.

## Data Records

The CN-OBEE dataset is publicly available for download from figshare^37^, and consists of eight comma-separated value (CSV) files. Electric power data were placed in a separate file to facilitate the analysis of electricity use. These files can be classified into the following three types.

### Room data file

There are six composite data files for the six main rooms, including five categories of measurement variables: dry bulb temperature, relative humidity, air pressure, window state, and occupant presence. Notably, the number of data columns for each variable is different owing to the different number of window sensors in each room.

### Electric power file

A file with instantaneous electric power data is available for all appliances. This file can be used for power analysis of each appliance, but can also be used to calculate the cumulative power consumption of all appliances in the family home.

### Outdoor weather file

There is an outdoor weather file, which includes data on the dry bulb temperature, relative humidity, atmospheric pressure, wind speed, wind direction, ground temperature, horizontal total solar radiation intensity, and horizontal diffuse solar radiation intensity.

The first column of each file is a regularized timestamp. Because data regularization follows 1-minute intervals, this amounts to 1440 data points per day (there are 24 data points per day in the outdoor weather data). The data collection period was 365 days, from May 31, 2021 to May 31, 2022. The details of the eight files are listed in Table 3.

**Table 3:** Files in dataset.

Number	File Name	Sampling Frequency	Number of Columns	Type of Files
1	Master_bedroom.csv	1 min	6	Room data file
2	Secondary_bedroom.csv	1 min	6	
3	Cloakroom.csv	1 min	5	
4	Home_office.csv	1 min	7	
5	Living_room.csv	1 min	7	
6	Kitchen.csv	1 min	6	
7	Power.csv	1 min	15	Electric power file
8	Outdoor_weather.csv	1 h	9	Outdoor weather file

## Technical Validation

This section discusses the technical effectiveness of the proposed dataset. First, a preliminary analysis of the missing data was conducted. Then, the accuracy of the data was validated.

### Missing data

**Fig. 5:**
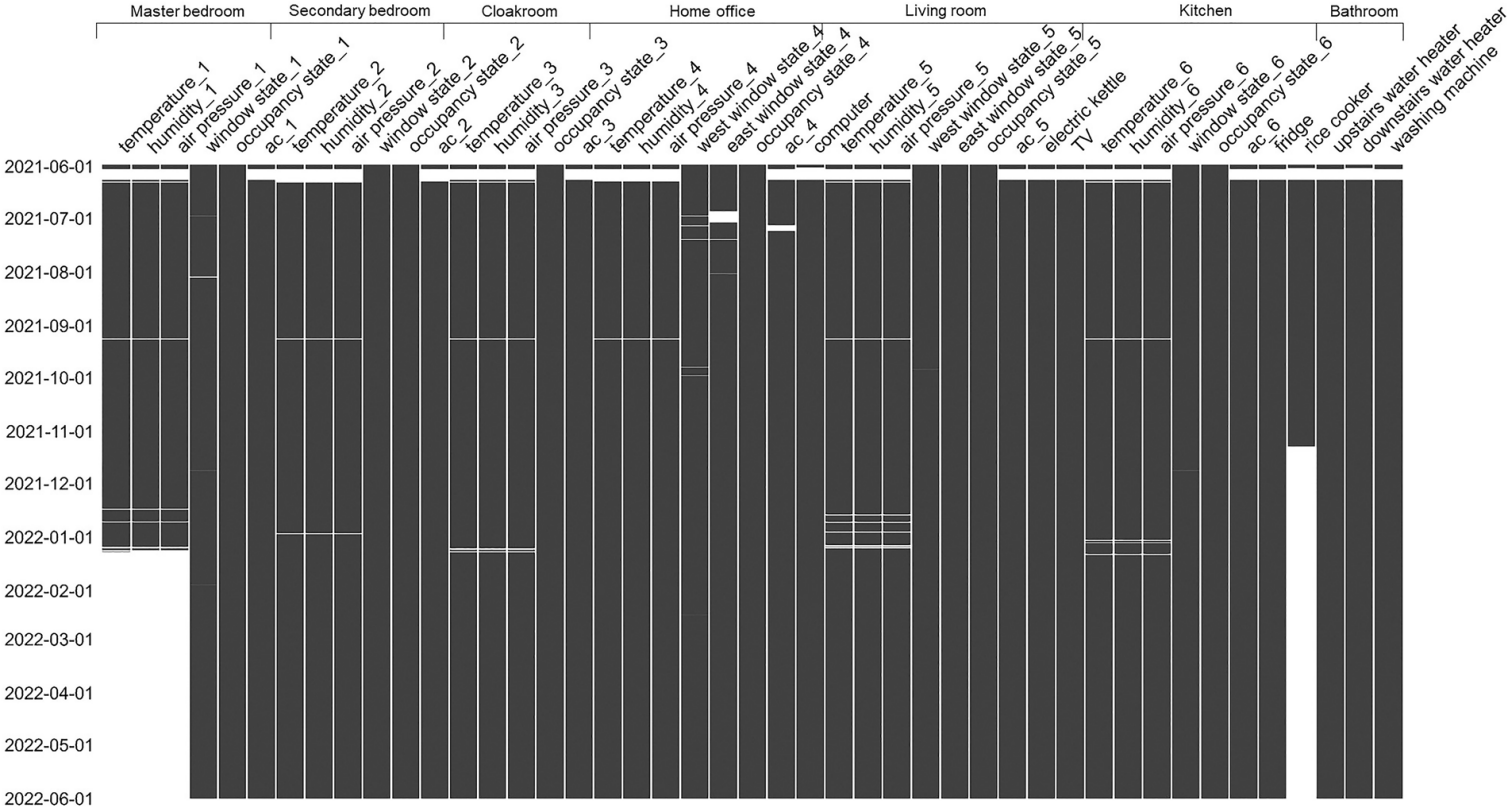
Time chart of missing data for each column in each room.

A detailed breakdown of the missing data in the relevant columns for each room is presented in Table 4. A time chart of the missing data in each column is shown in Fig. 5.

**Table 4:** Detailed breakdown of amount of missing data in relevant columns in each room.

Room	Column Name	Total	Missing Data Number	Missing Data Proportion
Master bedroom	dry-bulb temperature, relative humidity, and air pressure.	527041	219152	42%
	window state		1464	0%
	occupant presence		90	0%
	air conditioner #1		9110	2%
Secondary bedroom	dry-bulb temperature, relative humidity, and air pressure.	527041	12828	2%
	window state		0	0%
	occupant presence		34085	6%
	air conditioner #2		10423	2%
Cloakroom	dry-bulb temperature, relative humidity, and air pressure.	527041	12558	2%
	occupant presence		118	0%
	air conditioner #3		9097	2%
Home office	dry-bulb temperature, relative humidity, and air pressure.	527041	11269	2%
	west window state		1407	0%
	east window state		9947	2%
	occupant presence		23858	5%
	air conditioner #4		14162	3%
	computer		10273	2%
Living room	dry-bulb temperature, relative humidity, and air pressure.	527041	15462	3%
	west window state		93	0%
	east window state		245	0%
	occupant presence		0	0%
	air conditioner #5		9020	2%
	electric kettle		9017	2%
	TV		9022	2%
Kitchen	dry-bulb temperature, relative humidity, and air pressure.	527041	13192	3%
	window state		131	0%
	occupant presence		256	0%
	air conditioner #6		9094	2%
	fridge		9014	2%
	rice cooker		302397	57%
Bathroom 1	upstairs water heater	527041	9040	2%
	washing machine		9039	2%
Bathroom 2	downstairs water heater	527041	9793	2%

For the rice cooker in the kitchen, there are no data in the later period because the plug power sensor was unplugged. The remaining data gaps were caused by network interruptions, which led to data not being uploaded in time. For the temperature and humidity sensor in the master bedroom, the sensor was not found to be offline in time, owing to the authors’ negligence, resulting in a large amount of missing data.

### Indoor and outdoor thermal environment

**Fig. 6:**
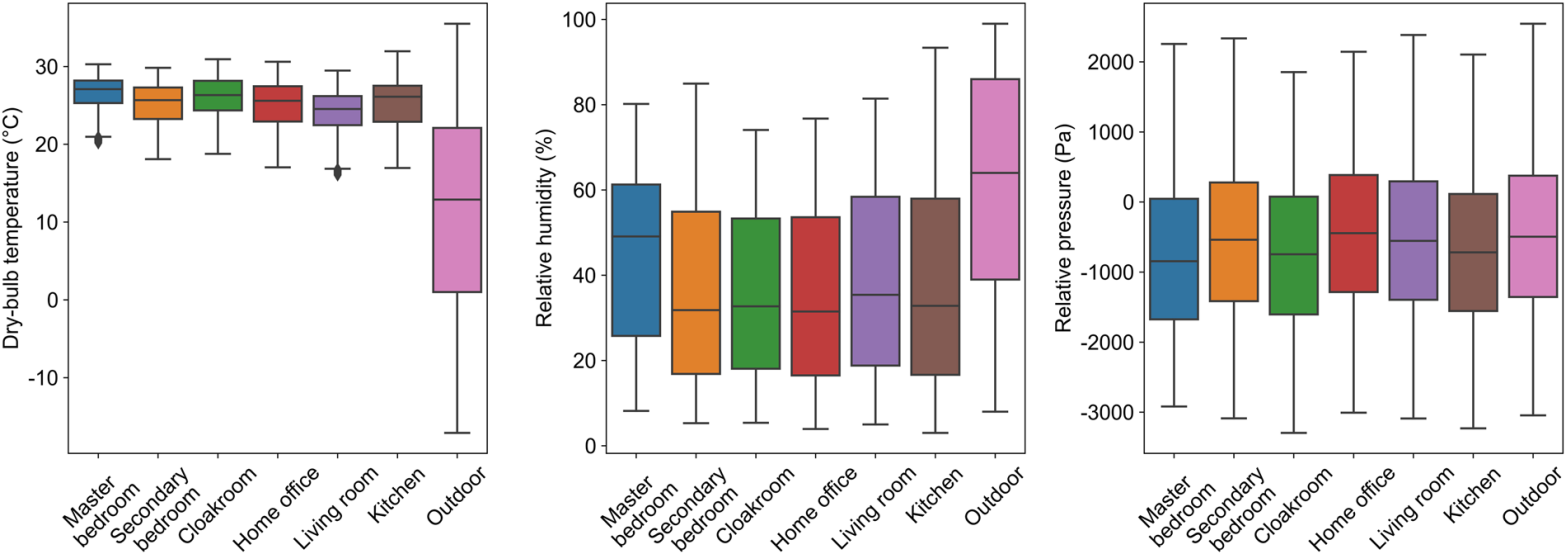
Box plot of dry-bulb temperature, relative humidity, and air pressure for each room and outdoor environment.

The dry-bulb temperature, relative humidity, and air pressure box diagram for each room and the outdoor environment over one year are shown in Fig. 6. As can be seen, the indoor dry-bulb temperature is between 15 °C and 30 °C, and the outdoor temperature is between -15 °C and 35 °C throughout the year. The indoor relative humidity is lower than the outdoor relative humidity, and mostly lies between 20% and 60%. The indoor thermal environment changes normally under the outdoor conditions, and extreme values do not exist. The outdoor weather data conform to the typical climate of Beijing.

### Occupant presence

The provided source data represent occupant motion and are used to determine the presence of occupants. Because this study considered a three-person family, most of the time, no more than three occupant simultaneous motions should be detected by all motion sensors. Owing to the occupants’ work routine, the occupant motion patterns on weekdays are different to those on weekends.

**Fig. 7:**
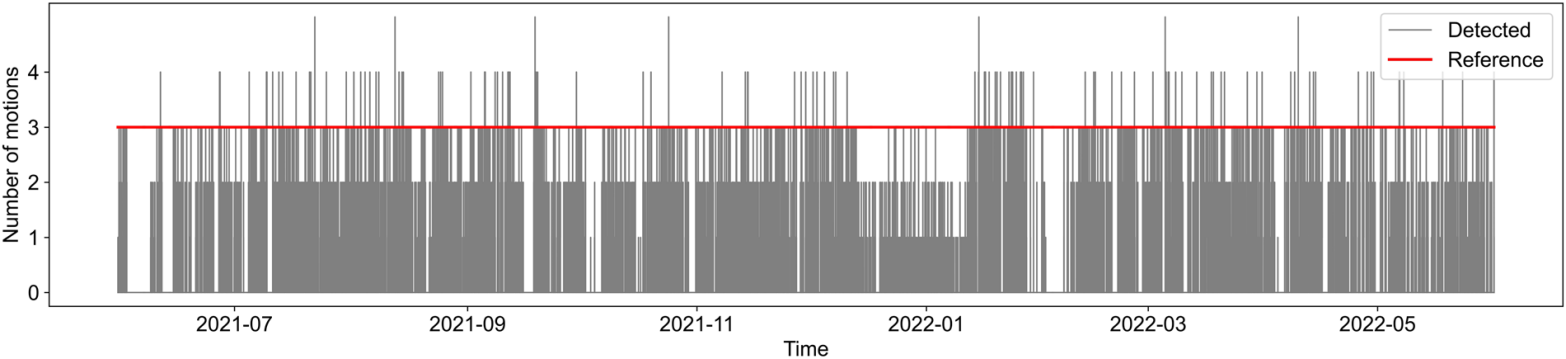
Number of occupant motions detected every minute in apartment.

Figure 7 shows the number of occupant motions detected every minute in the apartment. On some occasions, the number of occupant motions exceeds 3, with a proportion of 0.024%, which may reflect occupants moving into multiple rooms in one minute or guests visiting the apartment.

**Fig. 8:**
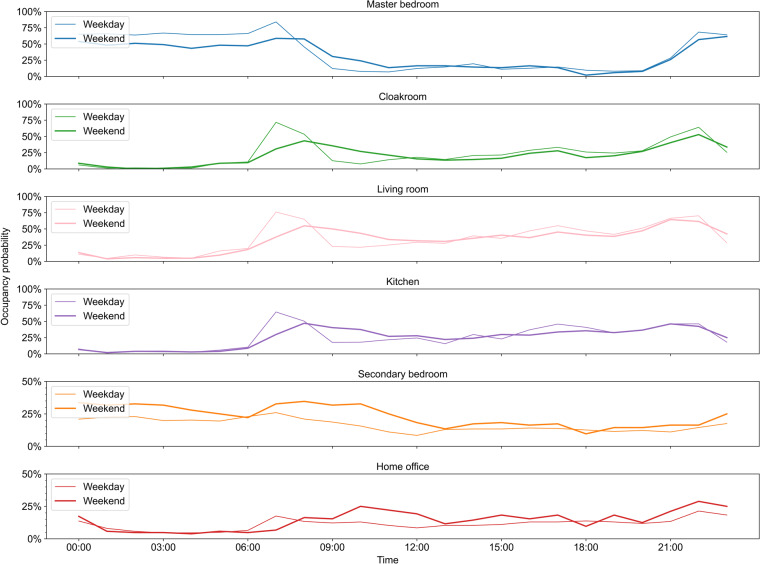
Hourly occupant presence probability for each room on weekdays and weekends.

Figure 8 shows the hourly occupant presence probability for each room on weekdays and weekends over one year. For the master bedroom, cloakroom, living room, and kitchen, there is a significant delay in the peak of occupancy probability between weekdays and weekends because the couple wakes up, eats breakfast, and has dinner later on weekends compared with weekdays. For the secondary bedroom and home office, the occupancy probability on weekends is greater than that on weekdays because the family’s adult offspring typically stays at school on weekdays and returns home on weekends.

### Relationship between window-opening and occupancy

**Fig. 9:**
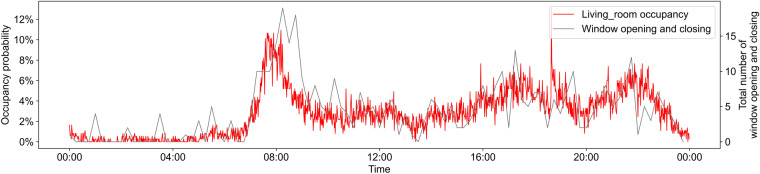
Number of window opening and closing actions and occupancy probability in living room.

Occupant presence is a prerequisite for the occurrence of window-opening behavior. The opening or closing of a window must be accompanied by occupant motion. Figure 9 shows the relationship between the number of window opening and closing actions and the occupancy probability in the living room. To make the graph smooth and clear, the number of window opening and closing actions was averaged over 15 minutes. The occupancy probability per minute was obtained by averaging the original motion data. As expected, window opening peaks after occupants move to the living room in the morning, and the two curves are in good agreement.

### Relationship between appliance power and occupancy

**Fig. 10:**
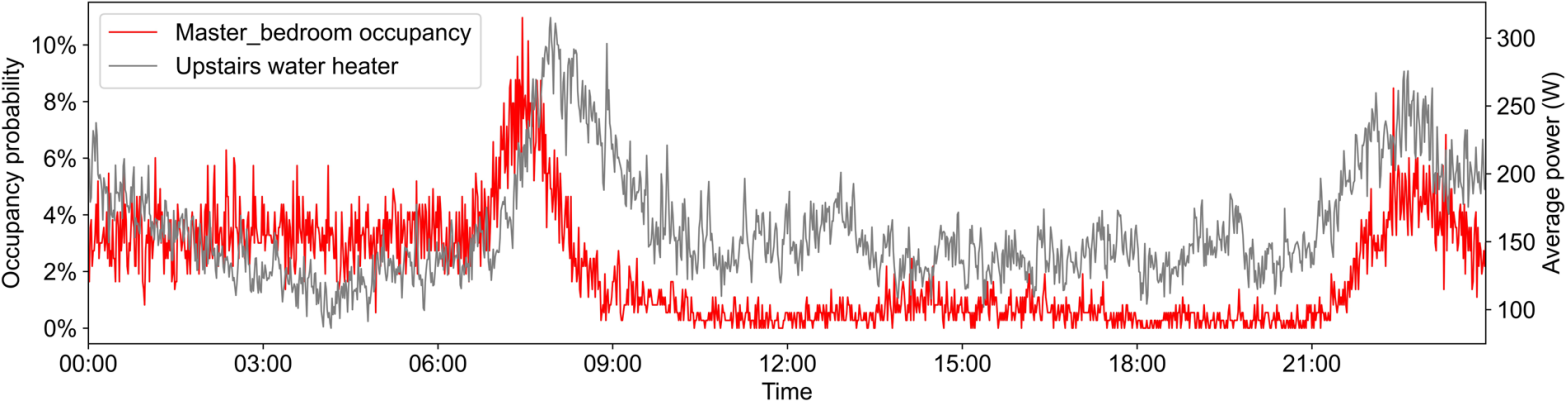
Per-minute average power of upstairs water heater and occupancy probability in master bedroom.

**Fig. 11:**
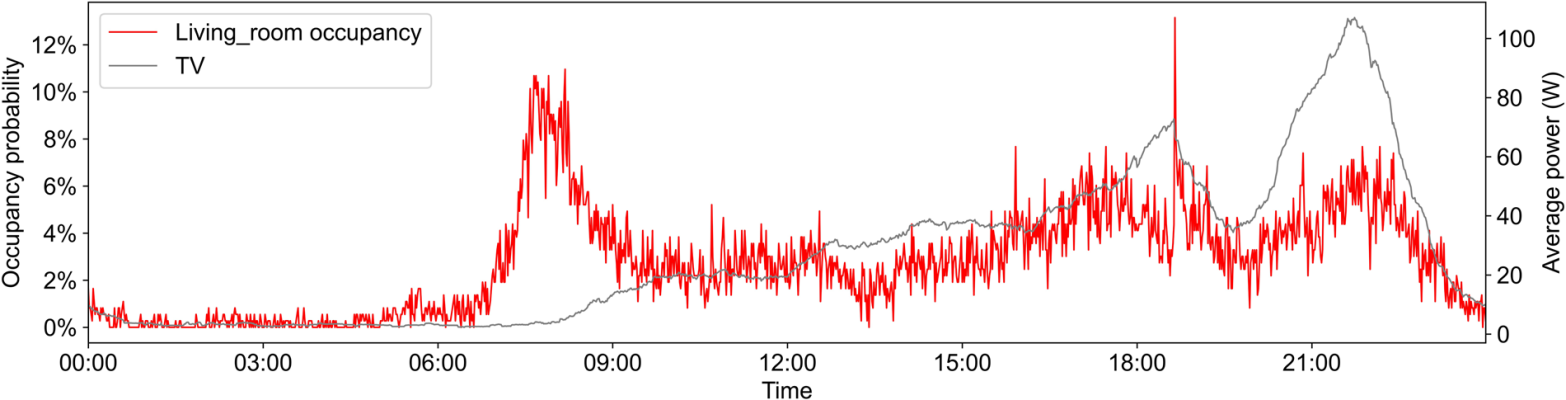
Per-minute average power of TV and occupancy probability in living room.

Occupant presence is also a prerequisite for the use of most electrical appliances. Figure 10 shows the relationship between the per-minute average power of the upstairs water heater and occupancy probability in the master bedroom. Figure 11 shows the relationship between the per-minute average power of the TV and occupancy probability in the living room. The couple mainly uses the upstairs water heater, therefore, its use peaks after the couple wakes up or before they go to sleep, which is consistent with their bathing habits. The TV usage peaks mainly during dinner or before sleep and matches well with the peak of occupancy probability in the living room.

### Peak power of appliances

Air conditioners and water heaters, which have high instantaneous power and energy consumption, are typical peak loads in residential buildings. Moreover, they have significant thermal storage capacity and can be used as free resources to participate in demand response.

**Fig. 12:**
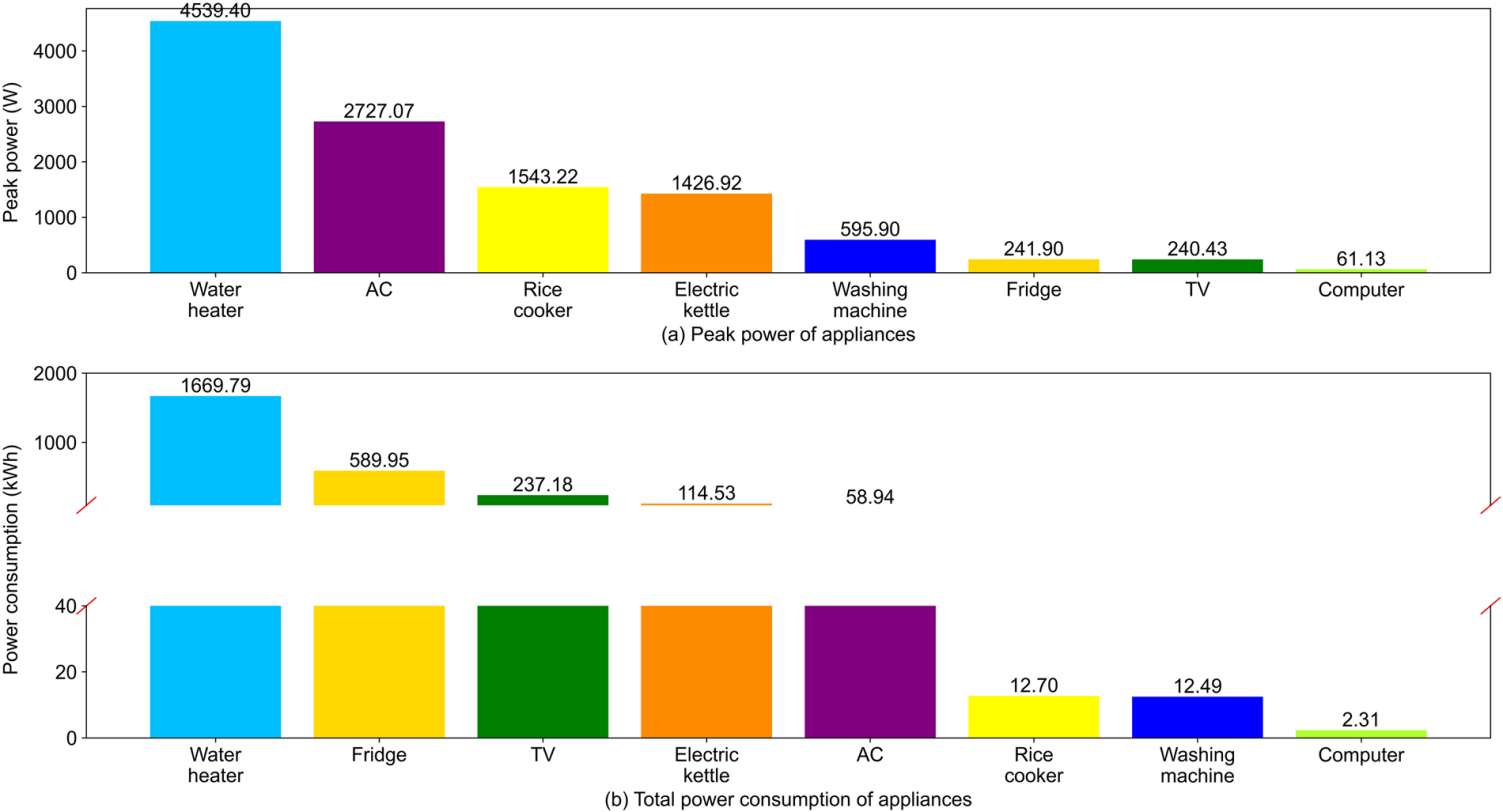
Peak power and total electricity consumption of appliances by category.

**Fig. 13:**
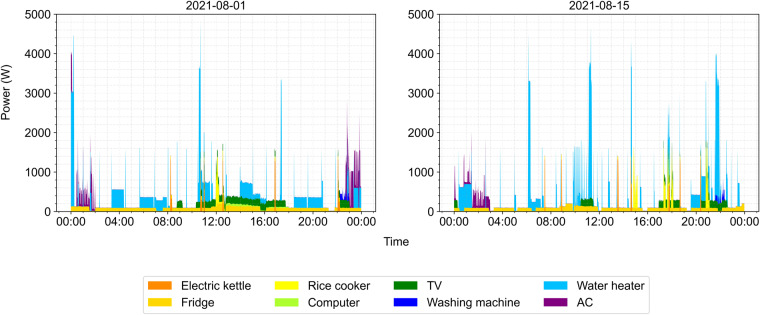
Instantaneous power of all appliance types in two typical summer days.

The peak power of appliances by category is shown in Fig. 12a. Figure 13 shows the instantaneous power of all appliance types in two typical summer days, including all above-mentioned electrical appliances, but excluding lighting and other small electrical appliances (mobile electronic equipment), which were not measured. As expected, the electric power peak of the family mainly results from the use of water heaters and ACs.

### Whole-year electricity consumption of appliances

The whole-year electricity consumption of appliances by category is shown in Fig. 12b. As can be seen, ACs do not have large total consumption on the annual time scale because they are only used in summer. The family’s total and AC electricity consumption were compared with those of other households in Beijing.

According to the China Building Energy Model^41,42^, the average annual electricity consumption of an urban household with three members and high-quality living standards in Beijing, is approximately 3300 kWh/a, excluding lighting. The reference value measured for the 120-m^2^ apartment of a single-family middle-class with five members in Beijing is approximately 2250 kWh/a^42^. In this dataset, the family’s electricity consumption, excluding lighting and other small electrical appliances, is approximately 2833 kWh/a, which is close to the reference value.

**Fig. 14:**
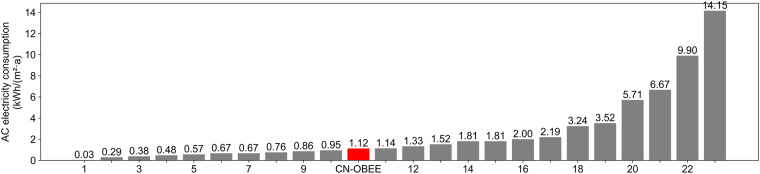
Electricity consumption of ACs in Beijing households.

Figure 14 shows the family’s whole-year AC electricity consumption in this dataset, and that of 23 Beijing households^41^, from lowest to highest. The data for 23 households were obtained in an apartment building with split ACs during 2006 and 2007. The results reveal that the difference in the AC electricity consumption can be more than 10-fold, owing to the different lifestyles and different AC usage patterns of different families. The total electricity consumption of ACs in this dataset is 1.12kWh/ (m^2^·a), which is close to the median of the 23 households (1.33kWh/ (m^2^·a)) and indicates that the family’s AC usage is at a normal level.

## Usage Notes

The presented dataset includes the electricity consumption of each appliance, indoor temperature and humidity, open or closed state of windows, and the state of occupancy for a typical urban family in Beijing, China, at room and appliance level and one-minute time intervals over an entire year. This dataset can support the following: electricity load shape analysis; electric power prediction; occupant behavior modeling and validation; building energy modeling and validation; thermal comfort analysis; suitability of energy-related measures; other types of research for residential buildings at household level.

In addition, this paper proposes an IoT-based data collection method, which is highly available to facilitate data collection. The proposed method has the following advantages: 1) miniscule wireless sensors consume little space and have little impact on home appearance; 2) low-power sensors support long-term measurement and do not require frequent battery replacement; 3) automatic, continuous, and remote real-time data collection; 4) the devices have low cost. The proposed method has good applicability to the measurement of room- and appliance-level information related to energy, the environment, and occupant behavior in residential buildings, and can be used to collect more data from households in the future, which will in turn help in responding to concerns on wide variations from household to household.

All data files in this study are available in CSV format with a total file size of 180 MB. The CSV data format is easy to use, and the file can be processed and analyzed by many popular programming languages, such as Python, Java, and C++. The Python pandas library is recommended for data processing and visualization.

### Supplementary information

Supplementary Table 1

## Data Availability

All data visualizations in this paper were created using Python and its publicly available libraries, including Pandas, NumPy, Matplotlib, Seaborn, and Missingno. A coding guide has been compiled in a Jupyter notebook and is provided along with the dataset in the Figshare repository.
