# Supplementary material for: Occupant behavior, thermal environment, and appliance electricity use of a single-family apartment in China
Source: Sci Data. 2024 Jan 11;11:65. doi: 10.1038/s41597-023-02891-9 (PMC10784530; doi:10.1038/s41597-023-02891-9)
Supplement: Supplementary file 1 — Supplementary Table 1 [file 41597_2023_2891_MOESM1_ESM.docx]

| Dataset | Location | Duration | No. of units | No. of appliance  instances | Measurements | | | | | | | | | | | | | | Sampling rate |
| --- | --- | --- | --- | --- | --- | --- | --- | --- | --- | --- | --- | --- | --- | --- | --- | --- | --- | --- | --- |
|  |  |  |  |  | Indoor Environment | Outdoor Environment | Window Status (ON/OFF) | Occupancy | | Electricity | | | | | | | | |  |
|  |  |  |  |  |  |  |  | Occupant  Number | Occupant Presence | Aggregate power | Appliance | | | | | | | |  |
|  |  |  |  |  |  |  |  |  |  |  | Electric Water Heater | Electric Air Heater | Central AC system | Split Air Conditioner | Kitchen and Cleaning Appliance | Entertainment | Electronics | Lighting |  |
| REDD^17^ | USA | 3-19 days | 6 houses | 10-24 per house |  |  |  |  |  | × |  | × | × |  | × |  | × | × | 15 kHz (Agg), 1 Hz (App) |
| BLUED^18^ | USA | 8 days | 1 house | 43 (on-off tag) |  |  |  |  |  | × |  |  |  |  |  |  |  |  | 12 kHz & 60 Hz (Agg) |
| FIRED^19^ | Germany | 52 days | 1 house | 21 | × |  |  |  |  | × |  |  |  |  | × | × | × | × | 8 kHz & 50 Hz (Agg), 2 kHz & 1Hz(App) |
| DEDDIAG^20^ | Germany | 3.5 years | 15 houses | 1-9 per house |  |  |  |  |  |  |  |  |  |  | × | × |  |  | 1 Hz |
| ENERTALK^21^ | Korea | 29-122 days | 22 houses | 1-7 per house |  |  |  |  |  | × |  |  |  |  | × | × |  |  | 15 Hz |
| ECD-UY^22^ | Uruguay | months-years | 111230 houses | 27 |  |  |  |  |  | × | × | × | × |  | × |  |  |  | 15 min. (Agg), 1min. (App) |
| Jens Hjort Schwee^23^ | Denmark | 44 days | 3 rooms | N/A | × |  |  | × | × |  |  |  |  |  |  |  |  |  | 1 min. |
| Margarite Jacoby^24^ | USA | weeks | 6 houses | N/A | × |  |  | × | × |  |  |  |  |  |  |  |  |  | 1 sec. (Occupancy), 10 sec. (Indoor) |
| Bing Dong 8^25^ | Canada | 3 months | 1 building | N/A | × | × | × |  |  |  |  |  |  |  |  |  |  |  | 15 min. |
| Bing Dong 11^25^ | USA | 1 year | 3 houses | N/A |  |  |  |  | × |  |  |  |  |  |  |  |  |  | irregular |
| Bing Dong 13^25^ | Poland | 1 year | 1 building | N/A |  | × |  |  |  |  |  |  | × |  |  |  |  |  | 1 day |
| Bing Dong 15^25^ | China | 6 months | 4 apartments | N/A |  | × | × |  |  |  |  |  |  |  |  |  |  |  | 20 min. (Outdoor), 5 min. (Window) |
| UK-DALE^26^ | UK | 36-655 days | 5 houses | 5-54 per house |  |  |  |  |  | × |  | × |  |  | × | × | × | × | 16 kHz & 1 Hz (Agg), 1/6 Hz (App) |
| AMPds2^27^ | Canada | 2 years | 1 house | 21 |  |  |  |  |  | × | × |  | × |  | × | × | × |  | 1 min. |
| SustDataED^28^ | Portugal | 10 days | 1 house | 17 |  |  |  |  | × | × | × |  |  |  | × | × | × |  | 12.8 kHz & 50 Hz (Agg), 1/2 Hz (App) |
| REFIF^29^ | UK | 2 years | 20 houses | 9 per house |  |  |  |  |  | × |  | × |  |  | × | × | × |  | 1/8 Hz |
| EMBED^30^ | USA | 14-27 days | 3 houses | 11-13 per house |  |  |  |  |  | × | × |  | × |  | × | × | × | × | 12 kHz & 60 Hz (Agg), 1 Hz (App) |
| fIEECe^31^ | USA | 9 months | 6 houses | 3 per house |  |  |  |  |  | × | × |  | × |  | × |  |  |  | 1 Hz |
| I-BLEND^32^ | India | 52 months | 7 buildings | N/A |  | × |  | × |  | × |  |  |  |  |  |  |  |  | 30min. (Outdoor), 10min. (Occupancy), 1 min. (Agg) |
| PRECON^33^ | Pakistan | 8 months | 42 houses | 1-34 per house |  |  |  |  |  | × |  |  | × |  | × |  | × |  | 1 min. |
| CN-OBEE^37^ | China | 1 year | 1 apartment | 14 | × | × | × |  | × |  | × |  |  | × | × | × | × |  | 1 min. |

**Supplementary Table 1** The details of the current publicly available residential datasets. (Sampling rate: Agg – Aggregate power; App – Appliance power).
